# Developing an Affordable Miniature 3D-Printed Wave Generator for Wave Energy Harvesting Application

**DOI:** 10.3390/mi15121500

**Published:** 2024-12-16

**Authors:** Yunzhong Wang, Damian Tohl, Anh Tran Tam Pham, Youhong Tang

**Affiliations:** College of Science and Engineering, Flinders University, Adelaide, SA 5042, Australia; steven.wang@flinders.edu.au (Y.W.); damian.tohl@flinders.edu.au (D.T.); anh.pham@flinders.edu.au (A.T.T.P.)

**Keywords:** laboratory automation, remote control, wave generator, modular concept design, 3D printing technology, expandable experimental platform

## Abstract

The development of low-frequency and low-amplitude wave energy harvesters has been limited by the lack of an affordable scientific evaluation platform, due to the high cost and land requirements of ground-based water channels. A 3D-printed modular wave generator, combined with the commercially available laboratory-sized wave channel, is proposed to address this. A stepper motor and an Arduino are employed as the driving source and controller. This system utilises motor parameters, such as rotational speed and number of travelled steps, to accurately control generated wave frequency and amplitude. By minimising costs and enhancing sustainability through 3D printing technology, only minor modifications are needed to adapt it to different water tank dimensions. The system can generate stable waves with frequencies from 1 Hz to 2 Hz and amplitudes from 1.5 cm to 7.1 cm under the current setting. The generated wave frequency and amplitude can be further customised by selecting faster stepper motors, as demonstrated in this study.

## 1. Introduction

The energy crisis is a significant challenge in the 21st century, which could deeply affect human civilisation development, particularly concerning electrical energy. The urgent need for alternative energy sources, driven by carbon neutrality regulations worldwide, has encouraged governments and organisations to focus on investigating clean energy solutions that do not contribute to carbon emissions [1]. Ocean wave energy has emerged as a focal point of interest due to the pressing challenges of the global energy crisis. With approximately 71% of the earth’s surface covered by oceans, ocean wave energy holds substantial potential as a sustainable and renewable energy solutions without the significant environmental effect and need for land [2,3]. Recently, researchers have focused on developing low-frequency and low-amplitude energy harvesters involving electromagnetic generators (EMG) and triboelectric nanogenerators (TENG) to harvest ocean wave energy [4,5,6,7,8,9,10,11]. However, the lack of an affordable evaluation platform has seriously limited the further development of ocean wave energy harvesters towards real applications, because current ground-based water channels are oversized, requiring massive amounts of land and being expensive to construct, making them hard to afford. Studying the dynamics of an ocean wave energy harvester under realistic ocean wave conditions is crucial for evaluating its output performance and contributing to the device’s design optimisation. Previous researchers have used commercial simulation software (Ansys AQWA 2021 R1) to find the best response frequency and amplitude to guide the design of ocean wave energy harvesters [12]. Nonetheless, finite element simulation often results in a lack of realism due to overfitting. As a result, researchers are attempting to modify existing devices to generate water waves to evaluate the performance of ocean wave energy harvesters. For instance, Wang et al. and Zhao et al. placed an electric water pump into a water tank to generate water waves. However, the generated waves were irregular and lacked variability in both frequency and amplitude because the waveform depended on the inlet and outlet water flow speed of the pump. This setup could only be used for demonstration rather than for scientifically evaluating the performance of the wave energy harvesters [13,14].

In 2022, Feng et al. and Wen et al. demonstrated a wave generation tank for a wave energy harvester. It utilised electric fans located on the side or bottom of the water tank to generate waves. This setup demonstrated stable wave frequency control but lacked the ability to change the wave amplitude due to the fixed contact area between the fan blades and water. Additionally, the generated waveform was closer to a swirl than a typical ocean wave, making it difficult to use for evaluating device performance [15,16]. In 2022, Shen et al. employed a motor-driven double-axle platform combined with a water tank positioned above this platform. Waves were generated through the back-and-forth motion of the platform. This setup allowed for simple amplitude and frequency control through the motor, but it faced limitations as the short water tank could not generate fully developed waves. Moreover, the motor-driven double-axle platform was also too expensive to afford [17]. In 2024, Zhou et al. used a self-developed wave generation device to demonstrate the performance of a wave energy harvester. However, due to the limited length of the wave tank, it could not fully develop low-frequency waves with long wavelengths [18]. Previous solutions not only lack variability in generated wave frequency and amplitude but also cannot provide a fully developed wave due to the limited length of the water tanks. A month later, Zhang et al. utilised a ground-based water channel system as an experimental platform to demonstrate a wave energy harvester. However, the large land requirements and high construction costs make ground-based water channels difficult to afford, as mentioned before [19]. Longer water channels can generate more stable and repeatable waves, which are crucial for evaluating the performance of wave energy harvesters. Consequently, a laboratory-sized water channel attracted our attention due to its significantly lower cost compared to a conventional ground-based water channel system, and it has a longer water tank compared to self-developed systems. Taking the HM 160 experimental flume from Gunt Hamburg (Barsbüttel, Germany) as an example [20], the entire system has a length of 5 m, which provides a decent length for a water tank with a long wavelength wave development, while also balancing land requirements and costs.

In this study, a modular wave generator, facilitating a commercially available laboratory-sized water channel, has been proposed to address this limitation. It was manufactured using 3D printing and laser cutting technologies with recyclable materials to satisfy sustainability requirements. This manufacturing approach can significantly reduce costs and minimize material waste when adjusting the water tank dimensions. The core driving unit of this device is based on a stepper motor and the Arduino programming platform, enhancing reproducibility. This research aims to overcome the limitations of current ground-based water channels and promote the development of a low-frequency ocean wave energy harvester. A comprehensive study of how motor parameters affect the generated waves in the water channel is presented here and verified by experimental results. Furthermore, a previously reported wave energy harvester (WD-TENG) is employed as a test device to demonstrate the capability of the developed wave generation device as an evaluation platform.

## 2. Results

### 2.1. Concept of Design and Functionality of Wave Generation Device

**Figure 1:**
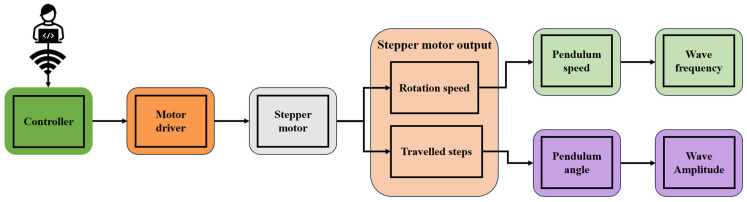
Workflow diagram of the wave generation device in this study.

The wave generation device utilises Bluetooth technology to enable remote wireless control of the generated wave amplitude and frequency. These parameters are controlled by a stepper motor in this design. The rotation speed of the stepper motor determines the generated wave frequency, while the number of travelled steps of the motor affects the generated wave amplitude, as shown in Figure 1. The demonstration of wireless control of the wave generation device is provided as shown in Video S1.

**Figure 2:**
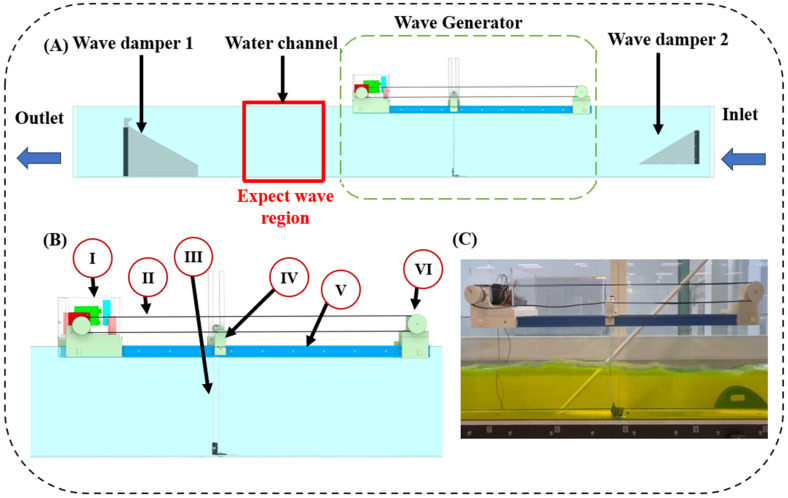
(**A**) Overview of the wave generation device, which involves two wave dampers and the commercial water channel; the red block represents the expected wave region, which will be utilised in future studies. (**B**) View of the six components that make up the wave generation device: (I) Motor driving unit, (II) transmission belt, (III) wave creation board, (IV) driving shaft, (V) T-track, and (VI) conveyor system balancer, and (**C**) photo of the actual wave generation device mounted in the water channel.

In this study, a commercial water channel (HM 160 Experimental flume, Gunt Hamburg, Germany) with dimensions of water channel length 500 cm, width 8.6 cm, and height 30 cm is used. Two multi-ladder-shaped wave dampers are employed to minimise the impact of reflected waves on newly generated waves. Wave Damper 1 and Wave Damper 2 are placed at the outlet and inlet of the water channel tank, respectively, as shown in Figure 2A. The inlet damper is designed with a gap between it and the bottom of the water channel to allow for free variations in water depth. The comparison between the setups with and without the damper is demonstrated in Video S2. The red block in Figure 2A is the region for stable generated waves and will be used in further analysis and demonstration.

The wave generation device follows a modular design concept to minimize waste when the water channel dimensions are modified, corresponding with sustainability goals. Based on the modular design, the wave generation device is composed of six core units: (I) motor driving unit, (II) transmission belt, (III) wave creation board, (IV) driving shaft, (V) T-track and (VI) conveyor system balancer as shown in Figure 2B.

(I) Motor driving unit: This is the core of the wave generation device and enables signal transmission between an endpoint laptop and a microcontroller via a Bluetooth module for remote control. By controlling stepper motor parameters, such as rotation speed, rotation direction, and number of travelled steps, this drives the timing belt and generates water waves through the wave creation board.

(II) Transmission belt: A timing belt transfers kinetic energy from the stepper motor through a 3D-printed pulley to the (III) wave creation board via the (IV) driving shaft.

(III) Wave creation board: It is driven by kinetic energy from the stepper motor via the (II) transmission belt and the (IV) driving shaft. The back-and-forth movement of the wave creation board generates waves. The board is placed inside the water channel tank and securely fixed to the bottom of the tank using screws from Gunt Hamburg.

(IV) Driving shaft: It is employed as a connector between the (II) transmission belt and (III) wave creation board, enabling the board to be driven by kinetic energy from the stepper motor. It is secured in the groove of the (V) T-track, and four stainless ball bearings are employed to reduce friction between the (IV) driving shaft and the (V) T-track.

(V) T-track: Two T-tracks are used to support the (I) motor driving unit and (VI) conveyor system balancer, forming the main body structure. This structure also provides support for the (IV) driving shaft when the device is mounted on the water channel tank. A 3D-printed lock is used to secure the positions of the (I) motor driving unit and (VI) conveyor system balancer on the T-track.

(VI) Conveyor system balancer: The core component of the conveyor system balancer is a stainless-steel ball bearing aligned with a 3D-printed toothed gear, together forming a transmission pulley. The conveyor system balancer is used to maintain the height of the (II) transmission belt and is positioned at the other end of the (V) T-track, enabling the horizontal conveyor system. This unit also uses a 3D-printed lock to secure its position.

The wave generation device installed on the water channel tank is shown in Figure 2C. Thanks to its modular design, the wave generation device offers versatile application possibilities, allowing it to integrate with laboratory-sized commercial water channels of varying dimensions with minimal modifications to the 3D-printed components.

### 2.2. The Working Mechanism of the Wave Generation Device

The generated waves are realised through the back-and-forth motion of the wave creation board driven by the stepper motor, through the adjustment of the rotation speed and the number of travelled steps of the motor taken to control the generated wave frequency and wave amplitude, respectively. Theoretically, the wave motion is represented as an approximate sinusoidal wave. The wave motion in the water channel system is described as follows:

(1) y=a× sin ⁡(bx−h)+k

where a, b, h, and k represent the wave amplitude, wavelength, horizontal phase shift, and vertical phase shift of the wave, respectively. Considering wave generation under static water conditions, where the water depth in the channel tank remains constant, the effect of vertical phase shift (k) can be ignored. Additionally, the use of two dampers effectively reduces the impact of horizontal phase shift (h), allowing it to be assumed as zero in this experimental setup.

To explain how the stepper motor is used to control the generated wave, two equations are employed to determine the force factors affecting the generated wave. The derivation of the force components along the X and Y axes is described as follows:

(2) $$f_{y}=F\times cos(\theta )$$

(3) $$f_{x}=F\times sin(\theta )$$

**Figure 3:**
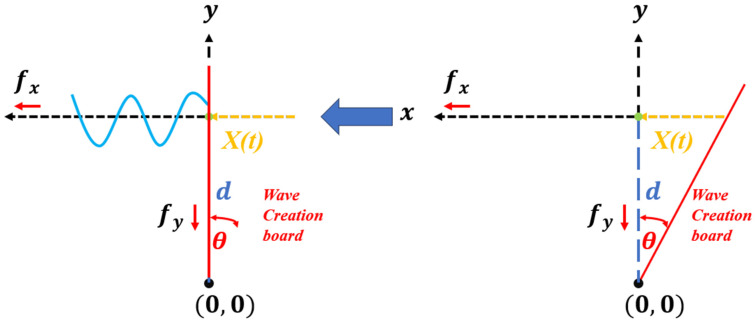
Schematic of the wave generating process, $f_{x}$ and $f_{y}$ represent the force component on x and y axes, respectively. d is the depth of water, and θ is the pendulum. x(t) represents the displacement of the wave creation board on the y-axes direction. Waves are generated when the wave creation board applies pressure to the contact area of the water.

Equation (2) describe the force component of the Y-axis of the wave creation board during wave generation, which is directly proportional to the water depth d as shown by the blue dashed line in Figure 3. Equation (3) represents the force component on the X-axis of the wave creation board corresponding to the pendulum θ, as shown by the yellow dashed line in Figure 3. Water depth d and pendulum θ will be the most important factors which will affect the generated wave amplitude. Pendulum θ is dependent on the number of travelled steps of the stepper motor, or named as the displacement of the wave creation board x(t). The water depth d will be adjusted by the HM 160 water channel system before wave generation. The period of one wave-generating cycle can be considered as the frequency of generated wave frequency f, and the frequency of the generated wave controlled by the motor rotation speed v. The back-and-forth movement of the wave creation board can be achieved by adjusting the rotation direction of the stepper motor, which applies pressure to the contact area of the water to generate waves.

(4) $$a=\frac{2\pi d}{L}x\left(t\right)=\frac{2\pi d}{\frac{v}{f_{w}}}S$$

Equation (4) is used to define the motor parameters that will affect the generated wave amplitude, where d, L, and $x\left(t\right)$ represent the water depth, wavelength, and displacement of the wave creation board, respectively [21]. Inserting the motor parameter into Equation (4), wavelength can be placed by $\frac{v}{f_{w}}$ where v and $f_{w}$ represent the motor rotation speed and the frequency of one wave generation cycle, respectively.

Consequently, there are three factors that will affect the amplitude of the generated wave, which are frequency $f_{w}$, which is dependent on motor rotation speed v; displacement of the wave creation board $x\left(t\right)$, which is dependent on the pendulum θ; and depth of water d.

(5) $$f_{w}=\frac{1}{\frac{2\cdot x\left(t\right)}{v}}=\frac{v}{2\cdot x\left(t\right)}$$

Equation (5) is utilised to define the frequency of the generated wave, where v represents the rotation speed, and $x\left(t\right)$ represents the displacement of the wave creation board named as the pendulum θ. Since the generated wave is caused by the back-and-forth movement of the wave creation board, there are two displacements of the wave creation board $2\cdot x\left(t\right)$ within one generation cycle. When the displacement of the wave creation board $x\left(t\right)$ is fixed, adjusting the motor rotation speed can control the generated wave frequency, f.

### 2.3. Analysis of the Generated Wave Under Variation of Water Depth

**Figure 4:**
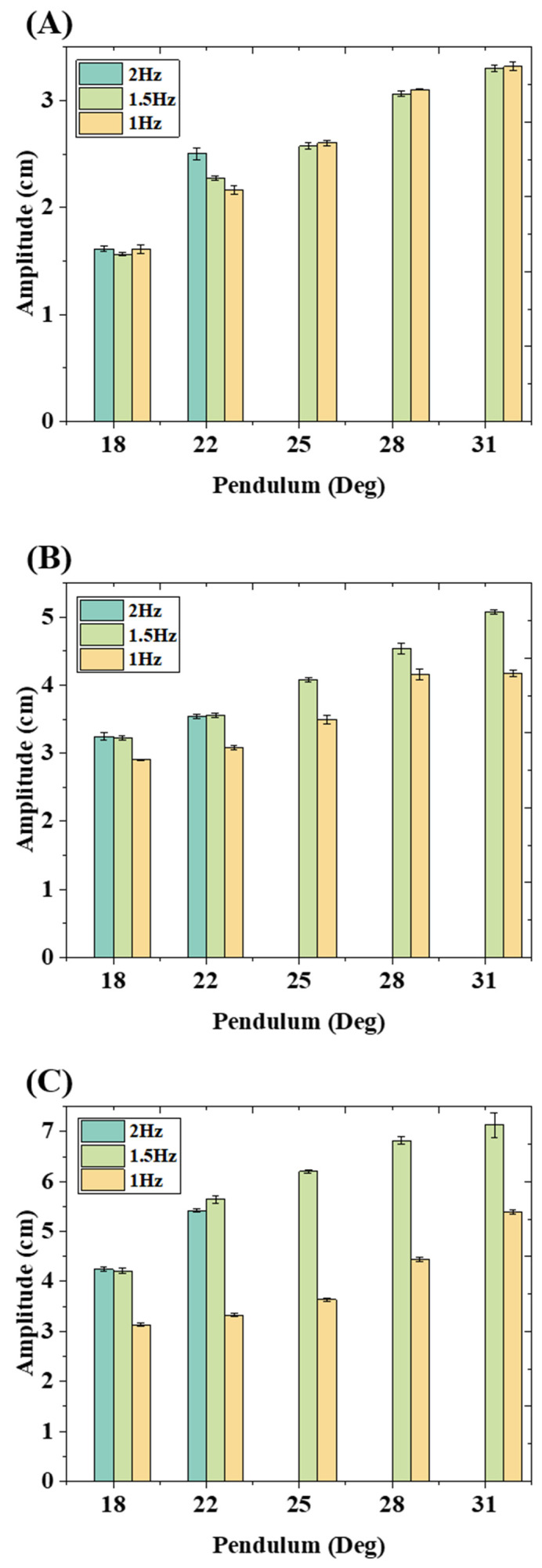
Detailed cylinder charts of generated wave amplitudes with error bars under (**A**) 10 cm depth, (**B**) 15 cm depth, and (**C**) 20 cm depth, respectively.

Figure 4 shows the mean amplitude of the generated wave with error bars for water depth 10 cm, 15 cm, and 20 cm, respectively. The tested pendulum angles are about 18°, 22°, 25°, 28°, and 31°, corresponding to 2500, 3000, 3500, 4000, and 4500 motor steps, respectively. The detailed pendulum is shown in Table S2. The maximum generated wave amplitude, approximately 7.1 cm, is observed at a frequency of 1.5 Hz, with a pendulum of 31° and a water depth of 20 cm. Additionally, the lowest amplitude of approximately 1.5 cm is recorded at a frequency of 1.5 Hz, with a pendulum of 18° and under the 10 cm water depth. However, the generated wave amplitude for all three frequencies, 1.0 Hz, 1.5 Hz, and 2.0 Hz, with a pendulum of 18° and water depth of 10 cm, was similar. When the pendulum increased from 18° to 31°, the generated wave amplitude demonstrated a significant direct proportional increase with the pendulum at various water depths. Furthermore, the results indicate that the increase in frequency, especially from 1.0 Hz to 1.5 Hz, does not significantly increase the generated wave amplitude for a water depth of 10 cm, compared with 15 cm and 20 cm water depth. The increase in frequency from 1.5 Hz to 2.0 Hz does not show a significant difference in amplitude for water depths of 15 cm and 20 cm, respectively. Therefore, the pendulum angle and the water depth are the most significant majority parameters that affect the generated wave amplitude. Moreover, the stability and repeatability of the wave generation device were verified under various water depths of 10 cm, 15 cm, and 20 cm. The maximum tolerance observed was 3.3% at a water depth of 20 cm, while the minimum tolerance was 2.0% at a water depth of 10 cm.

**Figure 5:**
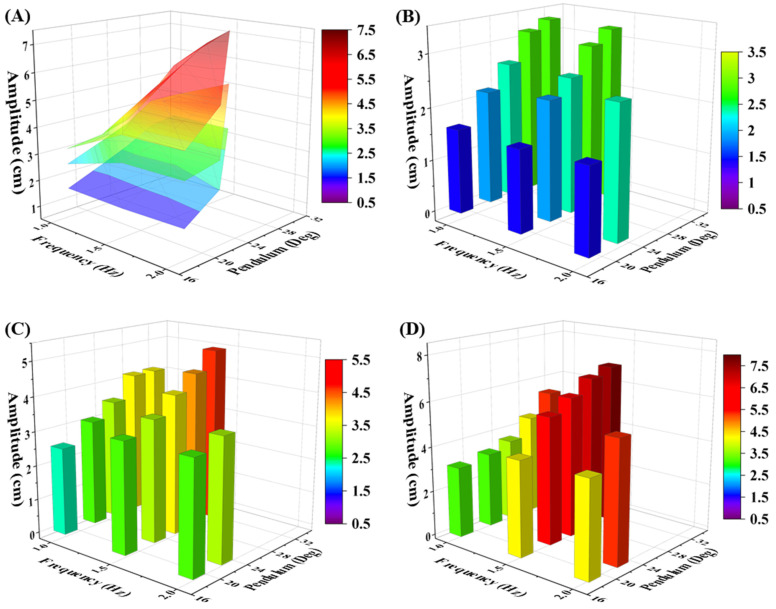
(**A**) Colormaps of the relationship between frequency, pendulum and amplitude under 10 cm, 15 cm, and 20 cm water depths. Detailed cylinder charts of generated wave amplitude under (**B**) 10 cm depth, (**C**) 15 cm depth, and (**D**) 20 cm depth.

Figure 5A depicts a 3D colormap that effectively illustrates the capacity of the wave generation device. The three tiers, from top to bottom, represent water depths of 20 cm, 15 cm, and 10 cm, respectively. The detailed trend of the change in amplitude for each water depth is shown in Figure 5B–D. Take the cylinder chart of 10 cm water depth, as shown in Figure 5B as an example; it demonstrates the trend of the change in amplitude of the generated wave with various pendulums. The change in pendulum has an obvious effect on the generated wave amplitude for each of the three water depths. A similar trend of change in amplitude has been observed at 15 cm and 20 cm water depths as shown in Figure 5C,D. Notably, the water depth d has the most significant influence on the generated wave amplitude when compared to frequency and pendulum. The experimental results verify the derived mathematical model. Moreover, although frequency f is one of the factors that affects the generated wave amplitude, as shown in Equation (5), the experimental results demonstrate that the generated wave amplitude is not significantly affected by frequency. To achieve higher wave frequency and amplitude in the future, a faster stepper motor, also manufactured by Trinamic (Hamburg, Germany), can be used to replace the current motor setup without modifying the connection layout. A maximum wave frequency of 8 Hz and a wave amplitude of 51 cm can be achieved with a faster motor and deeper water tank in the future. Details of the motor selection are provided in Note S1 and Tables S1 and S2 [22,23].

### 2.4. Analysis of the Wavelength of the Generated Wave Under Variations in Pendulum Angle

**Figure 6:**
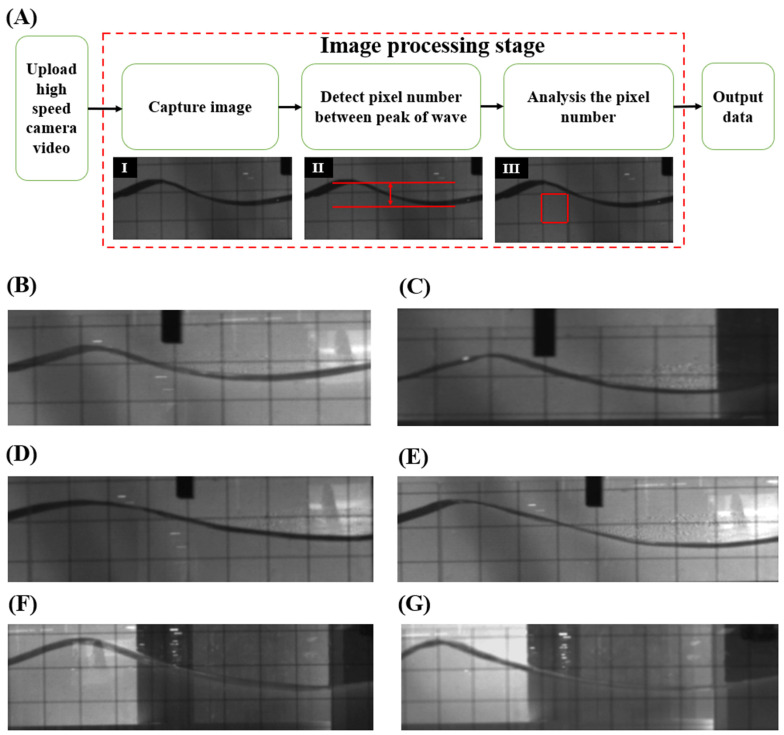
(**A**) A block diagram for the image processing steps to determine the mean value of the wave amplitude and the wavelength. Generated waves under 20 cm depth water, 40 cm wavelength under 2.0 Hz (**B**) 18°, and (**C**) 22°, 60 cm wavelength under 1.5 Hz (**D**) 18°, (**E**) 22°, (**F**) 28°, and (**G**) 31°.

A high-speed camera (FASTCAM NOVA S6, Photron, Tokyo, Japan) was employed to continuously record the motion of the generated waves in the water channel for 7.5 s. The high-speed camera we used captures thousands of photos over a 7.5 s period, to form a slow motion 3 min video. The video was then processed using a video editor to extract peak amplitude screenshots. Subsequently, an image processing method was applied to analyse the screenshots, as illustrated by the flowchart in Figure 6. Figure 6A(I) shows a captured image from the high-speed camera video, which was used to determine the peak-to-peak amplitude of the waveform in the next step. The number of pixels corresponding to the peak-to-peak amplitude was measured, as shown in Figure 6A(II). A 5 cm × 5 cm square grid backdrop was used to assist in the analysis of the wave amplitude. First, we counted the number of pixels from the top to the bottom of the square grid. Then, we calculated the pixel count of the peak-to-peak wave amplitude and converted it into metric units using the pixel count of the square grid, as shown in Figure 6A(III). Five generated waves were selected from the three-minute high-speed camera video to determine the mean value of the wave amplitude.

The wavelength of the generated wave for a water depth of 20 cm, under various frequencies and pendulums, is shown in Figure 6B–G. We applied the same image processing method to determine the wavelength. Figure 6B,C illustrates the wavelength of the generated wave at a frequency of 2.0 Hz and a pendulum of 18° and 22°, respectively, the results show that both waves have the same wavelength of approximately 40 cm. In addition, the wavelengths generated by a frequency of 1.5 Hz and pendulums under 18°, 22°, 28°, and 31° are shown in Figure 6D–G, respectively. The wavelength at a pendulum of 25° and a frequency of 1.5 Hz is shown in Figure S1D. They show a similar trend with a constant wavelength of approximately 60 cm. Furthermore, a constant wavelength of approximately 120 cm was generated for a frequency of 1.0 Hz with different pendulums as shown in Figure S1H–L. The wavelength of generated waves for a water depth of 15 cm and 10 cm are shown in Figures S2 and S3, respectively. Notably, the wave shows the same wavelength when observed under a water depth of 15 cm and 10 cm. An example of a generated wave is shown in Video S3. The relationship between the different pendulums, θ and rotation speeds, v, for different frequencies, f, are shown in Figure S4, which agrees with Equation (4), since wavelength, L, is equal to $\frac{v}{f}$. Therefore, the wave generation device shows the ability to generate a repeatable wave with a fixed frequency.

## 3. Demonstration

**Figure 7:**
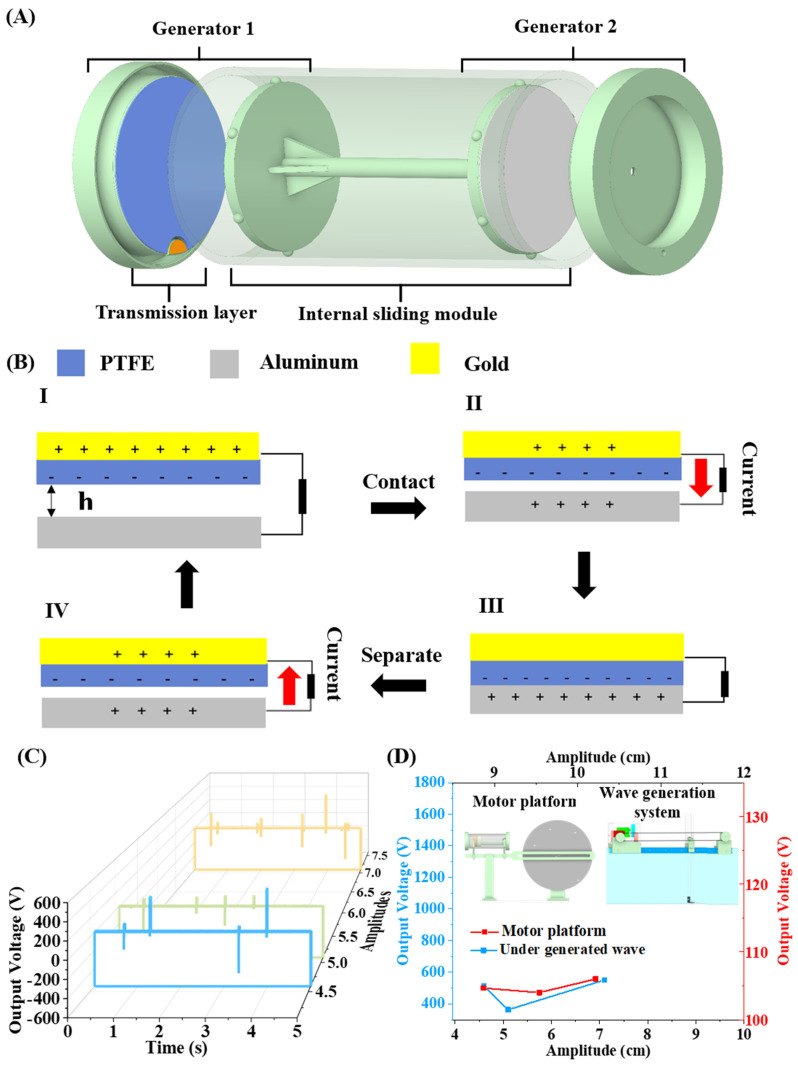
(**A**) The exploded view drawing of the WD-TENG, (**B**) the energy generation cycle of the WD-TENG, and (**C**) output performance of the WD-TENG under various generated wave amplitudes. The blue, green, and orange waveforms represent the generated waves with amplitudes of 4.6 cm, 5.1 cm, and 7.1 cm under a frequency of 1.5 Hz, respectively; and (**D**) the maximum output performance under the generated wave from current study comparing with the previous publication (Wang et al., 2022 [12]).

A previous published device named a wave-driven triboelectric nanogenerator (WD-TENG) was employed as test device to verify the feasibility of a modular design wave generation device as an evaluation platform for the low-frequency and low-amplitude wave energy harvester. WD-TENG utilises the coupling between contact electrification and the Triboelectric effect to convert kinetic energy into electricity. There is an internal sliding module inside the acrylic tube, used to place aluminium (Al) films (grey colour). Each bottom cap is equipped with a PTFE film (blue) and a small piece of copper used as an electrode (orange), as shown in Figure 7A. When one of the bottom caps faces an incoming generated wave, this side of the device is lifted, causing the internal sliding module to slide down to the other bottom cap. Initially, as shown in Figure 7B(I), the electron donor and acceptor are separated by a gap (*h*). When the materials make contact, electrons from the surface of PTFE flow to the surface of the aluminium, generating current, as shown in Figure 7B(II). The properties of electron donor and acceptor reverse after complete contact, as shown in Figure 7B(III); PTFE, the previous electron donor, becomes the acceptor due to the loss of electrons, while aluminium becomes the electron donor. If we separate both materials again, an inverse direction of current flow is generated, as shown in Figure 7B(IV). After one generation cycle is completed, the electron donor and acceptor return to their initial state. The dynamic of the WD-TENG under a generated wave of 7.1 cm amplitude with a 1.5 Hz frequency is shown in Video S4. As shown in Figure 7C, the WD-TENG responded four times to the generated wave with amplitudes of 4.6 cm and 5.1 cm. Once the amplitude was increased to 7.1 cm, the WD-TENG responded five times to the generated wave. Due to the higher amplitude, there was an increase in the gravitational force, allowing more kinetic energy to easily overcome the friction force between the internal sliding module and the acrylic tube.

Figure 7D shows the output voltage of the WD-TENG under actual waves generated by the proposed wave generation system compared to that of the previous motor-driven platform. The comparison results indicate that when the WD-TENG was tested with real waves created by the proposed wave generation device, it exhibited a similar trend to the previously published results but demonstrated significantly increased output performance due to the different dynamic motions of the WD-TENG. This observation emphasizes the importance of testing low-frequency and amplitude wave energy harvesters under actual water wave conditions. Additionally, this finding highlights that fully developed water waves have a noticeable effect on the dynamics of wave energy harvesters compared to previous results obtained from the motor-driven platform. The analysis results demonstrate the potential application of the wave generation device as a scientific evaluation platform for validating wave energy harvesters at the laboratory stage.

Due to the limitations of the current water channel, where the water in the tank is circulated by a pump, the use of saltwater or actual seawater could damage the pump and lead to failure. Therefore, the effect of different media has not been discussed in this study. Nevertheless, the wave generation device is designed to be employed as an evaluation platform for low-frequency and low-amplitude wave energy harvesters. While the medium in the water tank can influence the generated wave amplitude, it does not affect the output performance of the wave energy harvester under the same wave amplitude. Additionally, the dimensions of the water tank, such as water depth, will also affect the generated wave amplitude. A prediction of the generated wave amplitude of 51 cm with the current motor settings at a water depth of 40 cm is provided in Table S2.

## 4. Experimental Procedures

### 4.1. Fabrication and Functionality of the Component Consists of Wave Generation Device

#### 4.1.1. Motor Driving Unit

**Figure 8:**
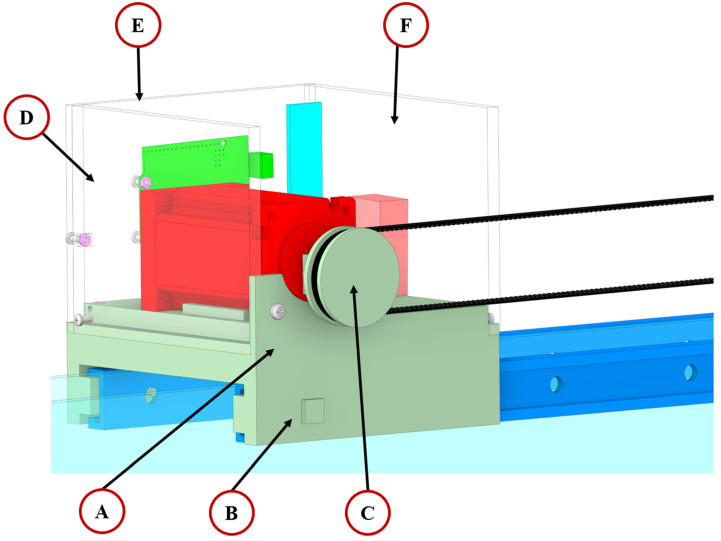
The detailed view of the motor driving unit. (A) Base, (B) locker, and (C) motor driving pulley are fabricated from polylactic acid (PLA) using 3D printing technology, as shown in green. (D) Left enclosure, (E) back enclosure, and (F) right enclosure are made from 4.5 mm thick transparent acrylic board using a laser cutting machine. The stepper motor is shown in red; the microcontroller is shown in green, the Bluetooth module is shown in bright blue, and the 9 V power supply is shown in pink.

The base of the motor driving unit (A), locker (B) and motor driving pully (C) are fabricated through a 3D printer by using polylactic acid (PLA) as shown in green components in Figure 8A–C. The bottom of the base (A) features notches designed for insertion into the grooves of the T-track. Additionally, two 3D-printed lockers (B) are employed to firmly secure the motor driving unit’s position on the T-tracks. The modular design allows the position of the motor driving unit to be adjusted as needed. The enclosure of the motor driving unit, comprising three pieces designed to safeguard the electronic components, is produced through a laser cutter machine with a 4.5 mm thickness transparent acrylic board, forming the white transparent part shown in Figure 8D–F.

The stepper motor (PD-1076, Trinamic, Hamburg, Germany), shown in red, was chosen as the kinetic energy source for the wave generation device. A microcontroller (Arduino UNO), shown in green, and a Bluetooth module (Dsd Tech Hc-05), shown in bright blue, are employed as the signal processor and signal receiver, respectively. The microcontroller converts Bluetooth signals from the endpoint laptop into digital signals for the motor driver, allowing the remote adjustment of the stepper motor parameters to control the generated wave frequency and amplitude. A 9 V power supply, shown in pink, is used to power the microcontroller. Thanks to the Bluetooth module, the wave generation device can be controlled wirelessly, eliminating the need for the operator to use a keypad or buttons.

#### 4.1.2. Conveyor System Balancer

**Figure 9:**
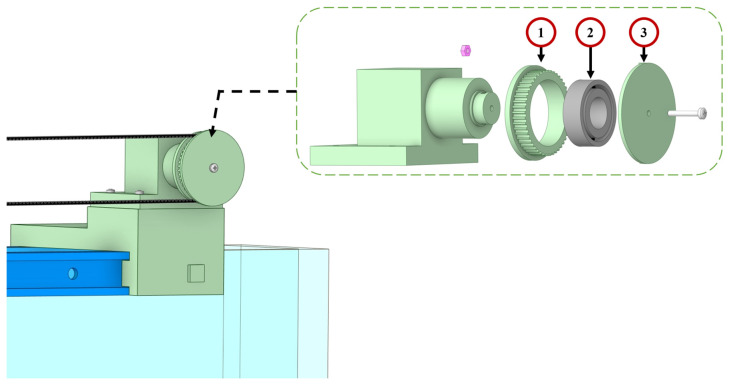
The close view of the conveyor system balancer, with a sub insertion which involves (1) 3D-printed pully, (2) steel ball bearing, and (3) transmission belt limiter. The 3D-printed components are shown in green.

The entire conveyor system balancer has been fabricated using 3D printing technology with polylactic acid (PLA) and assembled by M3 screws and nuts as shown in Figure 9. A ball bearing has been used to form a transmission pulley, as shown in the insertion figure. The 3D-printed pulley (1) with a 48-teeth gear is used to transfer the kinetic energy from the stepper motor, which is mounted on the ball bearing (2). A transmission belt limiter (3) is used to prevent system failure that could be caused by the timing belt slipping off the pulley (1). The conveyor system balancer also utilises a notched design concept similar to the motor driving unit. This notched design allows for the easy repositioning of the conveyor system balancer on the T-track by using 3D-printed lockers.

#### 4.1.3. Wave Creation Board

**Figure 10:**
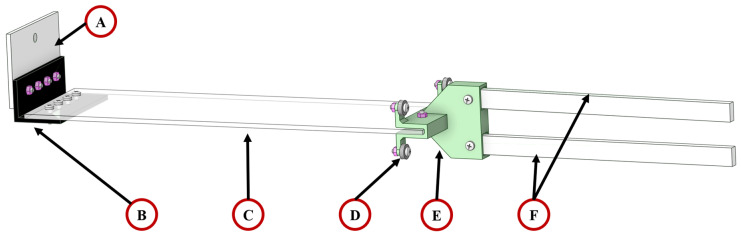
The detailed view of the wave creation board: (A) base board, (B) rubber connection piece, (C) wave generation board, (D) ball bearing, (E) shaft holder, and (F) wave creator shaft. The 3D-printed components are shown in green, and the white transparent pieces are acrylic components.

The components, consisting of a wave creation board, and excluding the rubber connection piece (B) and shaft holder (E), were fabricated from a white transparent acrylic board as shown in Figure 10. The shaft holder (E) was fabricated with 3D printing technology with polylactic acid (PLA). The base board (A) and wave generation board (C) are made from a 3 mm thick transparent acrylic board, and the wave creator shaft (F) is made from a 10 mm thick transparent acrylic board. The base fix board (A) is used to fix the commercial water tank system using the screws from Gunt Hamburg. A rubber piece (B) is used to connect the base fix board (A) and the wave creation board (C) by M3 screws and nuts. A shaft holder (E) is used to connect between the wave creation board (C) and two wave creator shafts (F). Four steel ball bearings (D) placed on each corner of the shaft holder (E) are used to reduce the friction between the wave creation board and glass water channel tank. Wave creators (F) are used to co-operate with the driving shaft to realise the back-and-forth movement of the wave creation board (C).

#### 4.1.4. Driving Shaft

**Figure 11:**
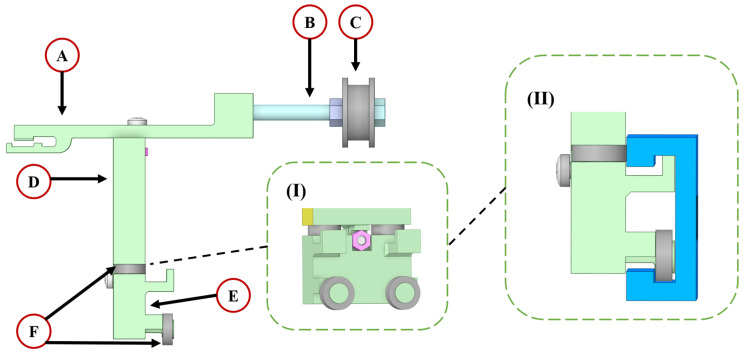
The detailed view of the driving shaft: (A) transmission belt connector, (B) metal shaft, (C) roller, (D) upper holder, (E) lower holder, and (F) ball bearing. The close view of the conjunction points (**I**) between the upper and lower holders; and the position of the four ball bearings on the T-track (**II**).

The transmission belt connector (A) is used to transfer kinetic energy from the stepper motor. A roller (C) is placed at the end of the metal shaft (B), secured with nuts, and inserted between the two wave creator shafts of the wave creation board. The holder of the driving shaft has been separated into two parts: the upper holder (D) and the lower holder (E). Both were fabricated using 3D printing technology with polylactic acid (PLA). They are aligned together using a screw and nut, as shown in Figure 11I. The designated positions for the four ball bearings (F) are illustrated in Figure 11II, with two positioned in the groove of the T-track and the other two in contact with the surface of the T-track. The purpose of utilising the ball bearings is to reduce the friction between the driving shaft and T-track.

### 4.2. Material and Components Used for Building Modular Concept Wave Generation Device

The parts of the wave generation device as shown in the green colour and transparent colour were fabricated by utilising a 3D printer (Monoprice Maker Ultimate 2+, Rancho Cucamonga, CA, USA) and a laser cutter (Rayjet 300EDU, Adelaide, Australia) to treat the transparent acrylic board and polylactic acid (PLA), respectively. The two T-tracks are used as the main body structure to set down units for the wave generation device. M3 screws and nuts were used to assemble the laser cut pieces and 3D-printed parts. A processor (Arduino UNO, Ivrea, Italy) was used to control the stepper motor’s driver (PD-1076, Trinamic, Hamburg, Germany). A Bluetooth module (Dsd Tech Hc-05) was used to transfer the digital signal to the stepper motor’s driver in order to generate the water wave. A 9 V battery dock (Jaycar, Sydney, Australia) was used to power the Arduino. A ball bearing E2.6003-2Z/C3 (SKF, Melbourn, Australia) was selected as the core component of the transmission pulley of the conveyor system balancer to balance the transmission belt position and transmit kinetic energy from the stepper motor. Eight stainless steel ball bearings (SMR106ZZ, RS PRO, Sydney, Australia) were used to reduce friction between the wave generation components and the glass water channel wall or the T-track, respectively.

### 4.3. Wave Energy Harvester Evaluation

The previously published wave-driven triboelectric nanogenerator (WD-TENG) (Wang et al., 2022 [12]) was used to validate the capabilities of the wave-generation device and verify its ability to serve as an experimental platform for the low-frequency wave energy harvesters. An oscilloscope (Keysight, InfiniiVision MSO-X 2004A, Santa Rosa, CA, USA) was connected to the TENG output to monitor the output electrical waveform to demonstrate the capacity of the wave generation device.

## 5. Conclusions

In this study, a modular concept wave generation device was proposed. Thanks to 3D printing technology and the modular design concept, the system requires only minor modifications to adapt to different water channel dimensions if needed in the future. This approach significantly saves costs and time while reducing material waste and enhancing material sustainability. In addition, the Arduino-based programming platform offers ample resources, making reproducibility more convenient. Moreover, the use of the Bluetooth module enables wireless remote control of the wave generation device, further enhancing its practicality. The relationship between the motor parameters and generated wave parameters has been derived by mathematical modelling and verified with results from the experiment. Based on the analysis results of the experiment, controllable and repeatable waves were successfully generated under various testing conditions from water depths of 10 cm to 20 cm. The frequency range of the generated waves was limited from 1.0 Hz to 2.0 Hz due to the maximum rotation speed of the currently selected stepper motor. However, a higher wave frequency and amplitude can be achieved by replacing the current stepper motor with a higher-speed one. The generated wave amplitude varies between 1.5 cm and 7.1 cm, demonstrating exceptional stability and repeatability with a maximum tolerance of 2.2%, 2.0%, and 3.3% for water depths of 10 cm, 15 cm, and 20 cm, respectively. A previously published ocean wave energy harvester was employed as a test device and successfully generated electricity under the generated waves. The demonstration results of the test device showed similar increasing trends compared to previously published results. The result confirms that the modular wave generation device offers a valuable solution for evaluating low-frequency and low-amplitude ocean energy harvesters, while also providing easier reproducibility. The effect of different media, such as saltwater or real seawater, needs to be understood in the future. Additionally, verifying whether the generated wave amplitude has a linear relationship with the dimensions of the water tank is worth investigating. The effect of different media, such as saltwater or real seawater, on the generated wave amplitude needs to be explored in the future. Additionally, it is worth investigating whether the generated wave amplitude has a linear relationship with the dimensions of the water tank. A predicted maximum wave amplitude of 51 cm can be achieved with a faster motor at a 40 cm water depth.

## Data Availability

Data will be made available on request.

## Supplementary Materials

The following supporting information can be downloaded at: https://www.mdpi.com/article/10.3390/mi15121500/s1, Figure S1. The wavelength of the generating wave is under 20 cm depth of water. Figure S2. The wavelength of the generating wave is under 15 cm depth of water. Figure S3. The wavelength of the generating wave is under 10 cm depth of water. Figure S4. The relationship between different Paddle angles (θ) and motor rotation speeds (v). Table S1. Comparison of step motor specifications to achieve higher wave frequency and amplitude. Table S2. Comparison of the generated wave amplitude between the experiment and prediction under 20 cm water depth and 1.5 Hz frequency. Video S1. Demonstration of wireless control of the modular concept wave generation device. Video S2: Comparison of generated waves with dampers and without dampers. Video S3: Demonstration of generated wave under 15 cm water depth with 1.5 Hz frequency and 24.69°. Video S4: Demonstration of the dynamic of WD-TENG under generated wave.

## References

[1] Australian Government (2023). Climate Active, Climate Active Carbon Neutral Standard 2023. https://www.dcceew.gov.au/climate-change/climate-active/.

[2] Melikoglu M. (2018). Current status and future of ocean energy sources: A global review. Ocean Eng..

[3] Khaligh A., Onar O.C. (2010). Energy Harvesting: Solar, Wind, and Ocean Energy Conversion Systems.

[4] Wang Z.L., Jiang T., Xu L. (2017). Toward the blue energy dream by triboelectric nanogenerator networks. Nano Energy.

[5] Liu L., Shi Q., Ho J.S., Lee C. (2019). Study of thin film blue energy harvester based on triboelectric nanogenerator and seashore IoT applications. Nano Energy.

[6] Wen X., Yang W., Jing Q., Wang Z.L. (2014). Harvesting broadband kinetic impact energy from mechanical triggering/vibration and water waves. ACS Nano.

[7] Wang W., Cao J., Zhang N., Lin J., Liao W.-H. (2017). Magnetic-spring based energy harvesting from human motions: Design, modeling and experiments. Energy Convers. Manag..

[8] Chen J., Yang J., Li Z., Fan X., Zi Y., Jing Q., Guo H., Wen Z., Pradel K.C., Niu S. (2015). Networks of triboelectric nanogenerators for harvesting water wave energy: A potential approach toward blue energy. ACS Nano.

[9] Li X., Tao J., Wang X., Zhu J., Pan C., Wang Z.L. (2018). Networks of high performance triboelectric nanogenerators based on liquid–solid interface contact electrification for harvesting low-frequency blue energy. Adv. Energy Mater..

[10] Liu W., Li Y., Tang H., Zhang Z., Wu X., Zhao J., Zeng L., Tang M., Hao D. (2024). The nexus of sustainable fisheries: A hybrid self-powered and self-sensing wave energy harvester. Ocean Eng..

[11] Zhang X., Zhang H., Zhou X., Sun Z. (2022). Recent advances in wave energy converters based on nonlinear stiffness mechanisms. Appl. Math. Mech..

[12] Wang Y., Pham A.T.T., Han X., Du D., Tang Y. (2022). Design and evaluate the wave driven-triboelectric nanogenerator under external wave parameters: Experiment and simulation. Nano Energy.

[13] Wang X., Niu S., Yin Y., Yi F., You Z., Wang Z.L. (2015). Triboelectric nanogenerator based on fully enclosed rolling spherical structure for harvesting low-frequency water wave energy. Adv. Energy Mater..

[14] Zhao X.J., Kuang S.Y., Wang Z.L., Zhu G. (2018). Highly adaptive solid–liquid interfacing triboelectric nanogenerator for harvesting diverse water wave energy. ACS Nano.

[15] Wen H., Yang P., Liu G., Xu S., Yao H., Li W., Qu H., Ding J., Li J., Wan L. (2022). Flower-like triboelectric nanogenerator for blue energy harvesting with six degrees of freedom. Nano Energy.

[16] Feng J., Zhou H., Cao Z., Zhang E., Xu S., Li W., Yao H., Wan L., Liu G. (2022). 0.5 m triboelectric nanogenerator for efficient blue energy harvesting of all-sea areas. Adv. Sci..

[17] Shen F., Zhang D., Zhang Q., Li Z., Guo H., Gong Y., Peng Y. (2022). Influence of temperature difference on performance of solid-liquid triboelectric nanogenerators. Nano Energy.

[18] Zhou J., Tang H., Zeng L., Zhang Z., Zhao J., Li A., Kong L., Tang M., Hu Y. (2024). A self-powered and self-sensing wave energy harvesting system for the sea-crossing bridge. Mater. Today Nano.

[19] Zhang C., Yang S., Dai X., Tu Y., Du Z., Wu X., Huang Y., Fan J., Hong Z., Jiang T. (2024). Hybridized triboelectric-electromagnetic nanogenerators for efficient harvesting of wave energy for self-powered ocean buoy. Nano Energy.

[20] Gunt Hamburg HM 160 Experimental Flume 86 × 300 mm. https://www.gunt.de/en/products/hydraulics-for-civil-engineering/hydraulic-engineering/open-channel-flow/experimental-flume-86x300mm/070.16000/hm160/glct-1:pa-148:ca-179:pr-595/.

[21] Galvin C.J. (1964). Wave-Height Prediction for Wave Generators in Shallow Water.

[22] Element 14 TRINAMIC/ANALOG DEVICES PD60-4-1076. https://au.element14.com/trinamic/pd60-4-1076/stepper-motor-driver-1-ph-2-8a/dp/2921448/.

[23] Element 14 TRINAMIC/ANALOG DEVICES PD86-3-1180-CANOPEN. https://au.element14.com/trinamic/pd86-3-1180-canopen/stepper-motor-2-ph-5-5a-7n-m/dp/2902251#/.

