# Supplementary material for: Developing an Affordable Miniature 3D-Printed Wave Generator for Wave Energy Harvesting Application
Source: Micromachines (Basel). 2024 Dec 16;15(12):1500. doi: 10.3390/mi15121500 (PMC11678848; doi:10.3390/mi15121500)
Supplement: Supplementary file 1 [file micromachines-15-01500-s001.zip › micromachines-3335107-supplementary/Supplemental - revised.pdf]

Supplementary Materials

Developing an Affordable Miniature 3D-Printed Wave Generator for Wave Energy Harvesting Application

Yunzhong Wang, Damian Tohl, Anh Tran Tam Pham and Youhong Tang *

College of Science and Engineering, Flinders University, Adelaide, SA 5042, Australia; steven.wang@flinders.edu.au (Y.W.); damian.tohl@flinders.edu.au (D.T.); anh.pham@flinders.edu.au (A.T.T.P.)

* Correspondence: youhong.tang@flinders.edu.au; Tel.: +61-8-82012138

Table of Contents

Figure S1. The wavelength of the generated wave is under 20 cm depth of water.

Figure S2. The wavelength of the generated wave is under 15 cm depth of water.

Figure S3. The wavelength of the generated wave is under 10 cm depth of water.

Figure S4. The relationship between different Paddle angles (θ) and motor rotation speeds (v).

Note 1. Selection of step motor to achieve higher wave frequency and amplitude.

Table S1. Comparison of step motor specifications to achieve higher wave frequency and amplitude.

Table S2. Comparison of the generated wave amplitude between the experiment and prediction under 20 cm water depth and 1.5 Hz frequency.

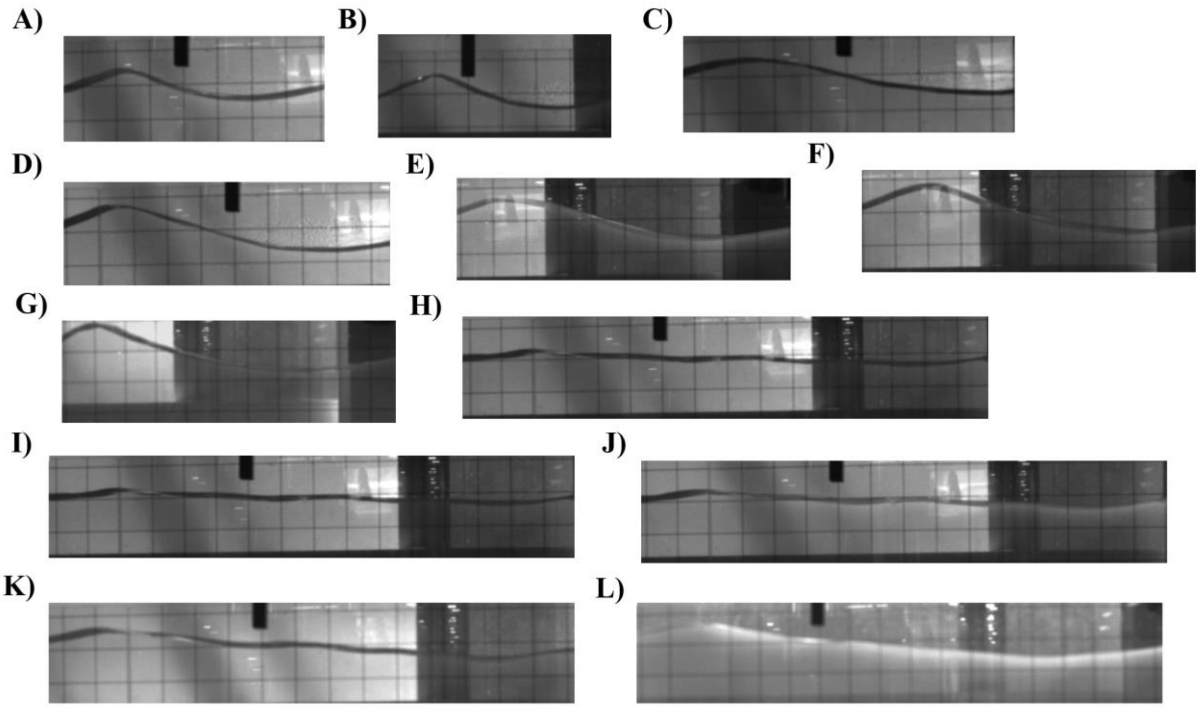

Figure S1. The wavelength of the generated wave is under 20 cm depth of water. A) 18 ° with 2 Hz, B) 22 ° with 2 Hz, C) 18 ° with 1.5 Hz, D) 22 ° with 1.5 Hz, E) 25 ° with 1.5 Hz, F) 28 ° with 1.5 Hz, G) 31 ° with 1.5 Hz, H) 18 ° with 1 Hz, I) 22 ° with 1 Hz, K) 25 ° with 1 Hz, J) 28 ° with 1 Hz, L) 31 ° with 1 Hz.

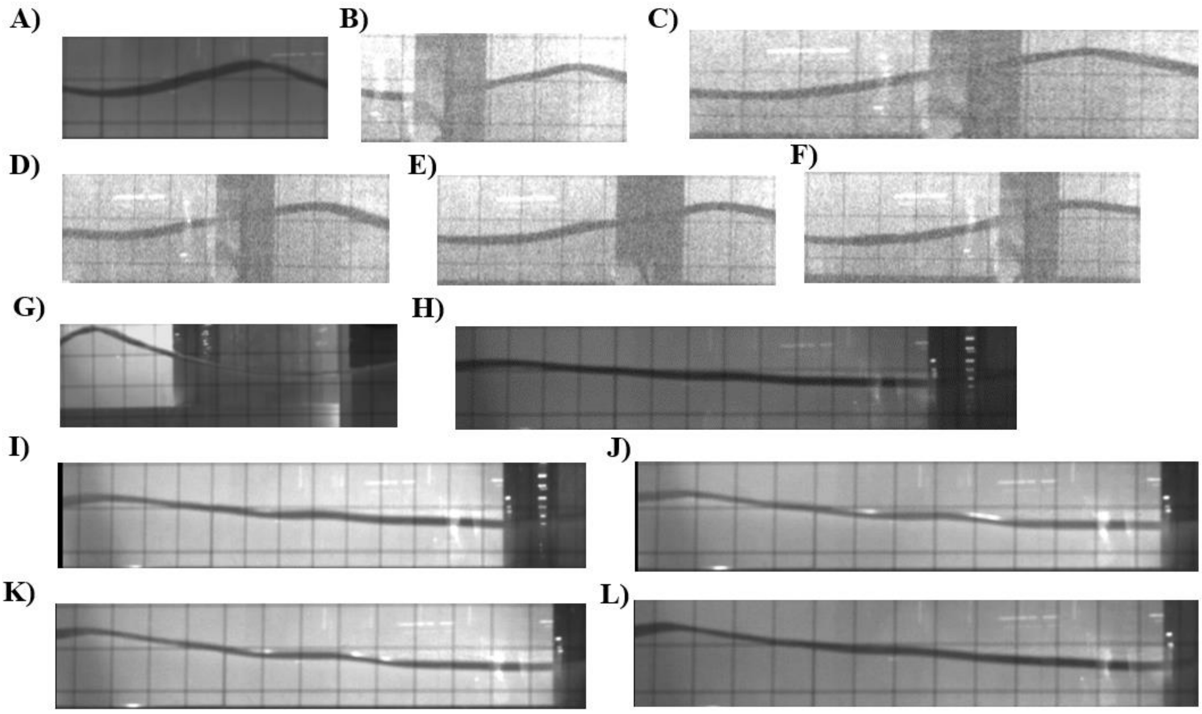

Figure S2. The wavelength of the generated wave is under 15 cm depth of water. A) 18 ° with 2 Hz, B) 22 ° with 2 Hz, C) 18 ° with 1.5 Hz, D) 22 ° with 1.5 Hz, E) 25 ° with 1.5 Hz, F) 28 ° with 1.5 Hz, G) 31 ° with 1.5 Hz, H) 18 ° with 1 Hz, I) 22 ° with 1 Hz, K) 25 ° with 1 Hz, J) 28 ° with 1 Hz, L) 31 ° with 1 Hz.

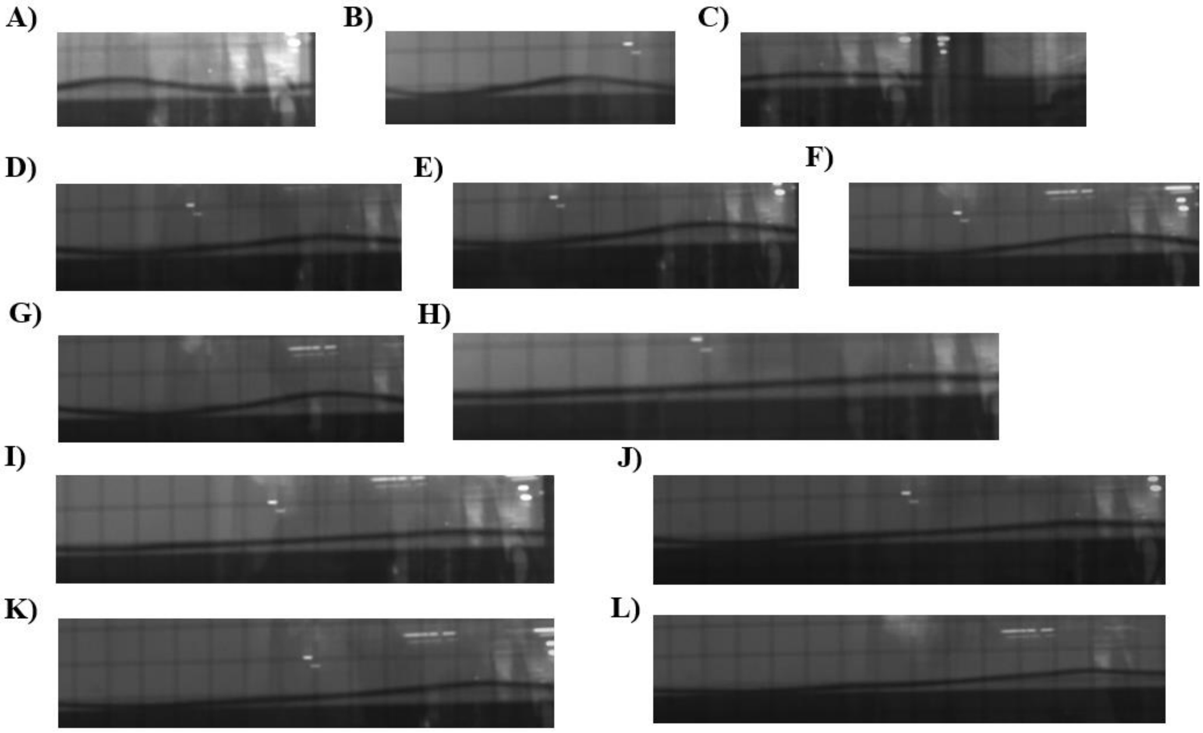

Figure S3. The wavelength of the generated wave is under 10 cm depth of water. A) 18 ° with 2 Hz, B) 22 ° with 2 Hz, C) 18 ° with 1.5 Hz, D) 22 ° with 1.5 Hz, E) 25 ° with 1.5 Hz, F) 28 ° with 1.5 Hz, G) 31 ° with 1.5 Hz, H) 18 ° with 1 Hz, I) 22 ° with 1 Hz, K) 25 ° with 1 Hz, J) 28 ° with 1 Hz, L) 31 ° with 1 Hz.

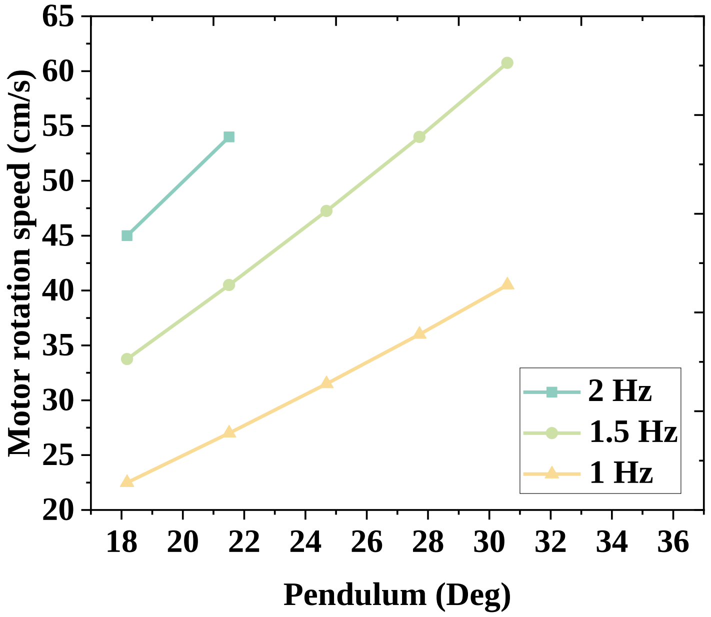

Figure S4. The relationship between different Paddle angles (θ) and motor rotation speeds (v) under various motor frequencies of 2 Hz, 1.5 Hz, and 1 Hz.

Selection of step motor to achieve higher wave frequency and amplitude.

As shown in Table S1, the specifications of the two stepper motors have been compared: the motor currently in use (PD60-4-1076) and another motor (PD86-3-1180) used to achieve higher wave frequency and amplitude. Both motors belong to the same series, have similar connection pins, and are manufactured by the same company, i. e., Trinamic (Germany). Therefore, there is no need to change the wiring layout; simply swapping the stepper motor allows for higher wave frequencies and amplitudes, significantly increasing convenience. Based on programming and analysis of experimental results, the maximum frequency achievable by the PD60-4-1076 is 2 Hz at a pendulum angle of 22°, due to its maximum rotation speed being limited to 1200 rpm. In comparison, the PD86-3-1180, with a maximum rotation speed of 5000 rpm, it can achieve a frequency at least four times higher, allowing wave frequencies to reach up to 8 Hz. The generated wave amplitude depends on the pendulum motion of the wave creation board. Using measurement data from a water depth of 20 cm and a frequency of 1.5 Hz, we derived a fitted equation (S1) to predict the generated wave amplitude at larger pendulum angles. The prediction results are shown in Table S2, where the wave amplitude can reach 25 cm at a pendulum angle of 50°. Additionally, based on experimental results and equation derivation, water depth has a more significant effect on wave amplitude compared to the pendulum angle. As shown in Figure 4 of the main text, the wave amplitude at a pendulum angle of 31° and a frequency of 1.5 Hz doubles when the water depth increases from 10 cm to 20 cm. Additionally, the generated wave is about one-third of the water depth. Therefore, if the water depth reaches 150 cm, the maximum generated wave amplitude is approximately 51 cm with 1.5 Hz and 50 pendulum.

$$\boldsymbol{Pridiction\ wave\ amplitude} = 0.00146981031998268x^3 - 0.122197924682524x^2 + 3.51665353674444x - 28.1493602662699 \quad \text{(S1)}$$

Table S1. Comparison of step motor specifications to achieve higher wave frequency and amplitude.

	PD60-4-1076 (Currently used) [1]	PD-3-1180 (For higher frequency and wave amplitude) [2]
Width (mm)	60	86
Height (mm)	60	86
Length (mm)	99	118
Weight (Kg)	1.4	2.8
Maximum rotation speed (rpm)	1200	5000
Price (AUD)	$450.99	$1280.75

Table S2. Comparison of the generated wave amplitude between the experiment and prediction under 20 cm water depth and 1.5 Hz frequency.

Experiment generated wave		Prediction generated wave	
Pendulum angle (°)	Wave amplitude (cm)	Pendulum angle (°)	wave amplitude (cm)
18	4.2	18	4.2
22	5.7	22	5.6
25	6.2	25	6.3
28	6.8	28	6.7
31	7.1	31	7.2
		33	7.7
		36	8.6
		38	9.8
		41	11.5

		43	13.5
		45	16.0
		46	18.8
		48	22.0
		50	25.4

Reference

1. Element 14. TRINAMIC / ANALOG DEVICES PD60-4-1076. https://au.element14.com/trinamic/pd60-4-1076/stepper-motor-driver-1-ph-2-8a/dp/2921448
2. Element 14. TRINAMIC / ANALOG DEVICES PD86-3-1180-CANOPEN. https://au.element14.com/trinamic/pd86-3-1180-canopen/stepper-motor-2-ph-5-5a-7n-m/dp/2902251#
